# Complications and Mortality after Surgeries in Patients with Prior Stroke Who Received General and Neuraxial Anesthesia: A Propensity-Score Matched Study

**DOI:** 10.3390/jcm11061490

**Published:** 2022-03-09

**Authors:** Yi-Ting Kao, Chuen-Chau Chang, Chun-Chieh Yeh, Chaur-Jong Hu, Yih-Giun Cherng, Ta-Liang Chen, Chien-Chang Liao

**Affiliations:** 1 Department of Anesthesiology, Shuang Ho Hospital, Taipei Medical University, New Taipei City 235, Taiwan; 19195@s.tmu.edu.tw (Y.-T.K.); stainless@s.tmu.edu.tw (Y.-G.C.); 2 Department of Anesthesiology, School of Medicine, College of Medicine, Taipei Medical University, Taipei 110, Taiwan; nekota@tmu.edu.tw (C.-C.C.); tlc@tmu.edu.tw (T.-L.C.); 3 Department of Anesthesiology, Taipei Medical University Hospital, Taipei 110, Taiwan; 4 Anesthesiology and Health Policy Research Center, Taipei Medical University Hospital, Taipei 110, Taiwan; 5 Department of Surgery, China Medical University Hospital, Taichung 404, Taiwan; b8202034@gmail.com; 6 Department of Surgery, University of Illinois, Chicago, IL 60607, USA; 7 Division of Neurology, Department of Internal Medicine, Shuang Ho Hospital, Taipei Medical University, New Taipei City 235, Taiwan; chaurjongh@tmu.edu.tw; 8 Department of Anesthesiology, Wan Fang Hospital, Taipei Medical University, Taipei 116, Taiwan; 9 Research Center of Big Data and Meta-Analysis, Wan Fang Hospital, Taipei Medical University, Taipei 116, Taiwan; 10 School of Chinese Medicine, College of Chinese Medicine, China Medical University, Taichung 404, Taiwan

**Keywords:** stroke, complications, mortality, neuraxial anesthesia, general anesthesia, surgery

## Abstract

Patients who previously suffered a stroke have increased risks of mortality and complications after surgeries, but the optimal anesthesia method is not fully understood. We aimed to compare the outcomes after surgeries for stroke patients who received general anesthesia (GA) and neuraxial anesthesia (NA). Using health insurance research data, we identified 36,149 stroke patients who underwent surgeries from 1 January 2008 to 31 December 2013. For balancing baseline covariates, the propensity-score-matching procedure was used to select adequate surgical patients who received GA and NA at a case–control ratio of 1:1. Multiple logistic regressions were applied to calculate adjusted odds ratios (ORs) with 95% confidence intervals (CIs) for postoperative mortality and complications between surgical patients with prior stroke who received GA and NA. Among the 4903 matched pairs with prior stroke, patients with GA had higher risks of pneumonia (OR 2.00, 95% CI 1.62–2.46), pulmonary embolism (OR 3.30, 95% CI 1.07–10.2), acute renal failure (OR 3.51, 95% CI 1.13–2.10), intensive care unit stay (OR 3.74, 95% CI 3.17–4.41), and in-hospital mortality (OR 2.02, 95% CI 1.16–3.51) than those who received NA. Postoperative adverse events were associated with GA in patients aged more than 60 years and those who received digestive surgery (OR 3.11, 95% CI 2.08–4.66). We found that stroke patients undergoing GA had increased postoperative complications and mortality after surgery compared with those who received NA. However, these findings need more validation and evaluation by clinical trials.

## 1. Introduction

Stroke remains a major global epidemic disease, affecting approximately 104 million individuals and accounting for 11.8% of all deaths worldwide [1,2]. The global estimation shows that the lifetime risk of stroke for people aged 25 years or older increased from 22.8% in 1990 to 24.9% in 2016 [3]. Patients with prior stroke have a higher risk of perioperative complications due to their pre-existing neurological disability, concomitant diseases, and vulnerability to the effects of anesthesia and surgery [4,5,6]. Anesthesia in surgery causes alterations in cardiac output, vascular resistance, blood pressure, and oxygen supply, which can prevent the development of postoperative complications in stroke patients [7].

It has been reported that NA (neuraxial anesthesia) for major truncal and lower limb surgery is associated with a lower risk of postoperative complications than GA (general anesthesia), including pulmonary complications, wound infection, and thromboembolism [8]. Additionally, NA is associated with reduced 30-day mortality risk for patients undergoing operations with moderate to high cardiac risk [9]. In addition, the previous study also demonstrated an increased risk of DNA damage after GA compared to NA [10].

Despite the growing burden of stroke on the health care system, few studies have evaluated the impact of different anesthetic techniques on the risk of postoperative complications in stroke patients. Most previous studies focused on patients undergoing carotid endarterectomy [11,12]; therefore, these investigations were restricted to acute management and intervention in acute ischemic stroke. Limited information was available regarding the risk of postoperative complications between patients who had a prior stroke with GA and NA. A Japanese study based on a small sample size found that stroke patients with GA did not have an increased risk of postoperative neurological complications compared with those who received NA (neuraxial anesthesia) [13]. Therefore, whether NA reduces postoperative complications in patients with cerebrovascular diseases remains unclear.

We used health insurance data from Taiwan to conduct a population-based study by propensity-score-matching methodology. The purpose of this study was to compare the risks of complications and mortality after surgeries in patients with prior stroke undergoing GA or NA. Based on previous evidence [8,9], we hypothesized that GA was associated with greater adverse outcomes after surgery than NA in stroke patients.

## 2. Methods

### 2.1. Source of Data

We designed this study by using research data from Taiwan’s National Health Insurance Program, and details of this database were described and evaluated in previous studies [6,14]. There was no direct contact with patients during data collection, and personally identifiable information was not included in the research data. Our study was reviewed and approved by the Joint Institutional Review Boards of Taipei Medical University (TMU-JIRB-201505055; TMU-JIRB-201705063; TMU-JIRB-201705065) and E-DA Hospital (EDA-JIRB-2017144), which also exempted informed consent form usage.

### 2.2. Study Design

We identified 36,149 patients with prior stroke aged ≥20 years who underwent surgeries (required GA or NA with hospitalization for more than 1 day) from 1 January 2008 to 31 December 2013 in Taiwan. Those surgical procedures that could only be performed with GA were excluded from this study. To eliminate confounding bias from baseline characteristics, we conducted propensity-score matching (case–control ratio = 1:1) to select eligible study subjects, so 4903 patients with GA and 4903 patients with NA were included in the final comparison.

### 2.3. Criteria and Definition

According to previous studies, the physician’s clinical diagnosis and the International Classification of Diseases, Ninth Revision, Clinical Modification (ICD-9-CM) were used to identify and define coexisting medical conditions and postoperative complications. Patients who had medical conditions within the preoperative 2 years were considered to have a history of disease in this study, including hypertension, diabetes, mental disorders, ischemic heart disease, chronic obstructive pulmonary disease, head injury, Parkinson’s disease, hyperlipidemia (hypercholesterolemia or hypertriglyceridemia), heart failure, liver cirrhosis, dialysis (hemodialysis or peritoneal dialysis), and alcohol-related illness. Previous visits for emergency care or hospitalizations within 2 years preoperatively were also identified as covariates in this study. The primary outcome of this study was 30-day in-hospital mortality, and the secondary outcomes were complications, including postoperative bleeding, pneumonia, septicemia, pulmonary embolism, urinary tract infection, deep wound infection, acute myocardial infarction, and acute renal failure recorded by the physician’s clinical diagnosis and ICD-9-CM.

Patients’ status of low income, history of medical visits (emergency care or hospitalizations within 2 years preoperatively), and types of surgery were also identified as covariates. We used quartiles of hospital volume to categorize the scale of hospitals as low (the lowest quartile), medium (the second and the third quartiles) and large (the highest quartile).

### 2.4. Statistical Analysis

Since there were several major differences between the GA and NA groups in baseline characteristics, further analysis of differences in the rate of postoperative adverse events between the two groups was undertaken. Patients with GA and patients with NA were matched according to a propensity score. The dependent variables in the propensity-score model included age, sex, low income, the volume of the hospital, types of surgery, number of emergency care visits, number of hospitalizations, hypertension, diabetes, mental disorders, ischemic heart disease, chronic obstructive pulmonary disease, head injury, Parkinson’s disease, hyperlipidemia, heart failure, liver cirrhosis, renal dialysis, and alcohol-related illness. Patients were matched by propensity score at a one-to-one ratio using the nearest-neighbor approach with no replacement and a caliper size of 0.1. A matched dataset containing 9806 patients (4903 in each group) was created.

The statistical analysis of the insurance data was performed using SAS software (version 5.1), and statistical significance was reported at *p* < 0.05. For continuous variables, the mean standard deviation (SD) was calculated by t tests as a summary statistic. For dichotomous variables, numbers and percentages were calculated by chi-square tests. Odds ratios (ORs) and 95% confidence intervals (CIs) were calculated as an approximation of the relative risk of postoperative complications and mortality in patients with different types of anesthetic modalities, taking general anesthesia as a reference.

## 3. Results

A total of 36,149 stroke patients were enrolled in the analysis (Table S1). After propensity-score matching (*n* = 9806), stroke patients were balanced in the two study groups in terms of demographic data, types of surgery, use of medical care, and medical conditions (Table 1).

After adjustment for the propensity scores (Table 2), stroke patients with GA had significantly higher odds for postoperative bleeding (OR 1.89, 95% CI 1.14–3.15), pneumonia (OR 2.00, 95% CI 1.62–2.46), septicemia (OR 1.62, 95% CI 1.35–1.95), acute renal failure (OR 3.51, 95% CI 1.13–2.10), pulmonary embolism (OR 3.30, 95% CI 1.07–10.2), deep wound infection (OR 2.27, 95% CI 1.15–4.50), intensive care (OR 3.74, 95% CI 3.17–4.41), and mortality (OR 2.02, 95% CI 1.16–3.51). Compared to stroke patients with NA, increases in the length of hospital stay (10.1 ± 13.7 vs. 7.2 ± 8.7 days, *p* < 0.0001) and medical expenditure (USD 3192 ± 3286 vs. 2229 ± 1739, *p* < 0.0001) were observed in stroke patients with GA. Risk of postoperative complications and mortality in stroke patients received surgery with general and neuraxial anesthesia (before matching) was showed in Table S2.

An analysis of data stratified by sex and age (Table 3) showed that GA was associated with postoperative adverse events in men (OR 1.82, 95% CI 1.54–2.15), women (OR 1.65, 95% CI 1.31–2.07), and patients aged 60–69 years (OR 1.74, 95% CI 1.24–2.44), 70–79 years (OR 1.80, 95% CI 1.44–2.25), and ≥80 years (OR 1.81, 95% CI 1.45–2.25). Further stratified analysis by medical conditions and medical services showed that the positive association between GA and postoperative adverse events was significant in stroke patients with zero (OR 2.10, 95% CI 1.60–2.75), one (OR 1.65, 95% CI 1.35–2.00), two (OR 1.50, 95% CI 1.12–2.00), and three (OR 2.62, 95% CI 1.45–4.74) medical conditions. The adjusted ORs of postoperative adverse events associated with GA for stroke patients who received musculoskeletal, digestive, and urinary surgery were 1.52 (95% CI 1.24–1.86), 3.11 (95% CI 2.08–4.66), and 1.71 (95% CI 1.38–2.12), respectively. The subgroup analyses (by types of stroke, the period of stroke occurrence, use of medications, rehabilitations, dementia, and pressure ulcer) for the risk of postoperative adverse events associated with GA are shown in Table S3.

## 4. Discussion

In this propensity-score-matched study, we found that GA administered to patients with prior stroke was associated with higher risks of pneumonia, wound infection, postoperative bleeding, admission to the intensive care unit, prolonged length of stay, and increased medical expenditures than NA. The association between GA and postoperative adverse events was significant in various subgroups.

Our study was strengthened by the application of propensity-score-matching procedures to adjust for multiple confounding factors. Most prior studies evaluated patients undergoing carotid endarterectomy and showed no significant difference in the events of stroke, myocardial infarction, or death between GA and NA [11,12,13]. For other types of surgical procedures, there is insufficient evidence regarding the advantage of one technique over the other in patients with cerebrovascular diseases. The benefits of NA in reducing postoperative complications have been extensively reported in hip, knee, abdominal, and vascular surgeries [8,9], although few studies have demonstrated the results pertaining to stroke patients.

Patients with prior stroke are prone to multiple comorbidities, which may further increase the postoperative risk in surgery [6,15]. A prior study showed that stroke-related comorbidities, including traumatic brain injury, dementia, pneumonia, and pressure ulcer, may further augment the increased postoperative risk in stroke patients undergoing surgery [6]. However, there remain few therapeutic strategies proven to be effective in improving postoperative outcomes for stroke patients. In this study, we evaluated the influence of preexisting comorbidities on stroke patients and showed that the increased postoperative risk associated with GA was independent of comorbidities.

Some possible explanations may be helpful for interpreting the risk of postoperative complications and mortality associated with GA. First, stroke patients have a higher risk of developing aspiration pneumonia due to dysphagia and loss of intact laryngeal cough reflex [16,17]. Studies have shown that the use of NA may reduce the risk of postoperative respiratory complications in patients undergoing surgery [18,19]. Muscle paralysis caused by GA may lead to atelectasis, which reduces lung volumes and oxygenation of the blood [20,21]. Second, our results suggested that NA was linked to lower risks of ICU stay and shorter length of hospital stay. Accumulating evidence indicates that NA is effective in improving postoperative organ function and ambulation, relieving postsurgical pain, decreasing opioid doses and related adverse effects, and facilitating postoperative recovery [22,23]. Third, NA may protect against surgical-site infection by moderating the inflammatory response to surgery and inducing vasodilation and consequent improvement in tissue oxygenation [14,24].

It is helpful to explain why stroke patients who received GA had an increased risk of acute renal failure. Studies have reported that NA reduces the risk of nephrotoxicity compared with GA, which alters renal blood flow and increases the risk of renal ischemia, reperfusion injury, and thereafter, acute kidney injury [25,26,27,28]. Reduced venous return in positive-pressure ventilation may compromise hemodynamic stability by increasing right atrium pressure, unbalancing blood volume distribution, and increasing vascular resistance [29,30], which may affect renal perfusion and cause acute kidney injury. In addition, a sympathetic block induced by epidural anesthesia does not significantly change renal blood flow, which may underlie the lower risk of acute kidney injury in patients with NA [31].

Our subgroup analyses revealed that the increased postoperative risk of GA was especially augmented in the subgroups of elderly patients undergoing digestive surgery. Studies have shown that old age is a significant predictor for poor functional recovery after ischemic stroke, independent of stroke severity and complications [32]. Furthermore, elderly patients are especially vulnerable to surgery-related physiological stress due to reduced functional reserve and potential homeostatic imbalance [33]. In addition to serving as a pain control technique, NA can facilitate mobilization and restore bowel function, thereby enhancing postoperative recovery in patients undergoing abdominal surgery [34,35]. Our results showed that NA may have a beneficial effect for the elderly and those with digestive surgery, offering important clinical implications for these subpopulations of stroke patients.

Based on our results, we suggest NA as the safer anesthetic technique for patients with prior stroke. However, stroke patients may have several concurrent pathologies contraindicating the use of NA, such as drug-induced coagulopathy (from antithrombotic agents), increased intracranial pressure, and intracranial hemorrhage [36]. Although our results showed that NA was associated with better postoperative outcomes for stroke patients, anesthesia providers should weigh this against potential bleeding risk for those with acquired coagulopathy on an individual basis. For patients with an absolute contraindication to neuraxial anesthesia, GA should be administered with close monitoring and optimal maintenance of cerebral perfusion to avoid recurrent cerebral ischemia or infarction. In addition, it is important to identify surgical patients at high risk of infection and renal failure prior to surgery. Prophylactic antibiotics and goal-directed hemodynamic therapy may reduce these adverse events after surgery for stroke patients [37,38].

There are some limitations to our study. First, our research data lacked information regarding physical examination (such as body mass index, heart murmur, breathing sound, heart rate, and blood pressure), biochemical measures (such as coagulopathy and blood sugar) and lifestyle (such as smoking, alcohol drinking, physical activity). Second, we had no data regarding the severity of stroke for each patient, such as the National Institute of Health Stroke Scale, which involved cerebral vascular territory, and modified Rankin Scale [39], which may affect postoperative prognosis and comorbidity. Third, detailed information on surgery and anesthesia, such as surgical and anesthetic procedures, perioperative medications, and physicians’ personal experiences and skills, was not available in this study. Another limitation is that we did not consider contraindications to NA in this study, such as coagulopathy, sepsis, increased intracranial pressure, and consciousness disturbance. Finally, confounding factors are possible, although our analyses controlled for a variety of potential confounding factors.

In conclusion, we found that patients undergoing NA had fewer complications, but a cause-and-effect assessment of this phenomenon is not possible due to the lack of adequate clinical data. Our findings provide an important implication to surgeons and anesthesiologists for optimal anesthetic management in this vulnerable population. Future randomized controlled trials are needed to validate the findings and elucidate their application.

## Figures and Tables

**Table 1:** Characteristics of stroke patients undergoing surgery with general and neuraxial anesthesia (after matching).

	NA (*n* = 4903)		GA (*n* = 4903)		*p*-Value
Sex	n	(%)	N	(%)	1.0000
Female	1934	(39.5)	1934	(39.5)	
Male	2969	(60.5)	2969	(60.5)	
Age, years					1.0000
20–29	12	(0.2)	12	(0.2)	
30–39	28	(0.6)	28	(0.6)	
40–49	138	(2.8)	138	(2.8)	
50–59	486	(9.9)	486	(9.9)	
60–69	982	(20.0)	982	(20.0)	
70–79	1908	(38.9)	1908	(38.9)	
≥3	1349	(27.5)	1349	(27.5)	
Low income					1.0000
No	4883	(99.6)	4883	(99.6)	
Yes	20	(0.4)	20	(0.4)	
Volume of hospital					1.0000
Low	1176	(24.0)	1176	(24.0)	
Medium	2125	(43.3)	2125	(43.3)	
High	1602	(32.7)	1602	(32.7)	
Types of surgery					1.0000
Musculoskeletal	2505	(51.1)	2505	(51.1)	
Peripheral vascular	7	(0.1)	7	(0.1)	
Digestive	717	(14.6)	717	(14.6)	
Kidney, ureter, bladder	1294	(26.4)	1294	(26.4)	
Delivery, CS, abortion	10	(0.2)	10	(0.2)	
Others	370	(7.6)	370	(7.6)	
Number of hospitalizations					1.0000
0	2306	(47.0)	2306	(47.0)	
1	1478	(30.1)	1478	(30.1)	
2	482	(9.8)	482	(9.8)	
≥3	637	(13.0)	637	(13.0)	
Number of emergency visits					1.0000
0	1940	(39.6)	1940	(39.6)	
1	1344	(27.4)	1344	(27.4)	
2	659	(13.4)	659	(13.4)	
≥3	960	(19.6)	960	(19.6)	
Coexisting medical conditions					
Hypertension	2136	(43.6)	2136	(43.6)	1.0000
Diabetes	969	(19.8)	969	(19.8)	1.0000
Hyperlipidemia	64	(1.3)	64	(1.3)	1.0000
Mental disorders	779	(15.9)	779	(15.9)	1.0000
Ischemic heart disease	365	(7.4)	365	(7.4)	1.0000
Heart failure	56	(1.1)	56	(1.1)	1.0000
COPD	349	(7.1)	349	(7.1)	1.0000
Liver cirrhosis	13	(0.3)	13	(0.3)	1.0000
Renal dialysis	16	(0.3)	16	(0.3)	1.0000
Alcohol-related illness	14	(0.3)	14	(0.3)	1.0000
Parkinson’s disease	67	(1.4)	67	(1.4)	1.0000
Traumatic brain injury	136	(2.8)	136	(2.8)	1.0000

COPD, chronic obstructive pulmonary disease; CS, cesarean section; GA, general anesthesia; NA, neuraxial anesthesia.

**Table 2:** Risk of postoperative complications and mortality in stroke patients undergoing surgery with general and neuraxial anesthesia (after matching).

	NA (*n* = 4903)		GA (*n* = 4903)		Outcome Risk	
Postoperative Outcomes	Events	%	Event	%	OR	(95% CI) †
30-day in-hospital mortality	19	0.4	38	0.8	2.02	(1.16–3.51)
Postoperative complications						
Pneumonia	146	3.0	276	5.6	2.00	(1.62–2.46)
Septicemia	206	4.2	322	6.6	1.62	(1.35–1.95)
Acute renal failure	68	1.4	103	2.1	3.51	(1.13–2.10)
Pulmonary embolism	4	0.1	13	0.3	3.30	(1.07–10.2)
Urinary tract infection	605	12.3	659	13.4	1.11	(0.98–1.26)
Deep wound infection	12	0.2	27	0.6	2.27	(1.15–4.50)
Acute myocardial infarction	13	0.3	21	0.4	1.62	(0.81–3.25)
Postoperative bleeding	23	0.5	43	0.9	1.89	(1.14–3.15)
ICU stay	205	4.2	658	13.4	3.74	(3.17–4.41)
Medical expenditure, USD ‡	2229 ± 1739		3192 ± 3286		*p* < 0.0001	
Length of hospital stay, days ‡	7.2 ± 8.7		10.1 ± 13.7		*p* < 0.0001	

CI, confidence interval; GA, general anesthesia; NA, neuraxial anesthesia; OR, odds ratio. † Adjusted for all covariates listed in Table 1. ‡ Mean ± SD.

**Table 3:** The stratified analysis in stroke patients undergoing surgery associated with adverse events.

			Adverse Events †			
		*n*	Events	Rate, %	OR	(95% CI) ‡
Female	NA	1934	141	7.3	1.00	(reference)
	GA	1934	217	11.2	1.65	(1.31–2.07)
Male	NA	2969	268	9.0	1.00	(reference)
	GA	2969	442	14.9	1.82	(1.54–2.15)
Age 20–59 years	NA	664	40	6.02	1.00	(reference)
	GA	664	56	8.43	1.45	(0.95–2.23)
Age 60–69 years	NA	982	62	6.3	1.00	(reference)
	GA	982	101	10.3	1.74	(1.24–2.44)
Age 70–79 years	NA	1908	146	7.7	1.00	(reference)
	GA	1908	242	12.7	1.80	(1.44–2.25)
Age ≥80 years	NA	1349	161	11.9	1.00	(reference)
	GA	1349	260	19.3	1.81	(1.45–2.25)
Low volume of hospital	NA	1176	127	10.8	1.00	(reference)
	GA	1176	202	17.2	1.79	(1.40–2.29)
Medium volume of hospital	NA	2125	175	8.2	1.00	(reference)
	GA	2125	292	13.7	1.82	(1.49–2.23)
High volume of hospital	NA	1602	107	6.7	1.00	(reference)
	GA	1602	165	10.3	1.63	(1.26–2.12)
Musculoskeletal surgery	NA	2505	176	7.03	1.00	(reference)
	GA	2505	256	10.2	1.52	(1.24–1.86)
Digestive surgery	NA	717	36	5.0	1.00	(reference)
	GA	717	98	13.7	3.11	(2.08–4.66)
Kidney, ureter, bladder surgery	NA	1294	172	13.3	1.00	(reference)
	GA	1294	264	20.4	1.71	(1.38–2.12)
Other surgery	NA	370	24	6.5	1.00	(reference)
	GA	370	38	10.3	1.68	(0.98–2.89)
0 medical condition	NA	1488	88	5.9	1.00	(reference)
	GA	1488	171	11.5	2.10	(1.60–2.75)
1 medical condition	NA	2186	197	9.0	1.00	(reference)
	GA	2186	300	13.7	1.65	(1.35–2.00)
2 medical conditions	NA	969	94	9.7	1.00	(reference)
	GA	969	132	13.6	1.50	(1.12–2.00)
3 medical conditions	NA	213	21	9.9	1.00	(reference)
	GA	213	44	20.7	2.62	(1.45–4.74)
≥4 medical conditions	NA	47	9	19.2	1.00	(reference)
	GA	47	12	25.5	1.53	(0.54–4.38)

CI, confidence interval; GA, general anesthesia; NA, neuraxial anesthesia; OR, odds ratio. † Adverse events included 30-day in-hospital mortality, pneumonia, septicemia, acute renal failure, pulmonary embolism, deep wound infection, postoperative bleeding. ‡ Adjusted for all covariates listed in Table 1.

## Data Availability

The data underlying this study is from the Health andWelfare Data Science Center. Interested researchers can obtain the data through formal application to the Health andWelfare Data Science Center, Department of Statistics, Ministry of Health andWelfare, Taiwan. Under the regulations from the Health and Welfare Data Science Center, we have made the formal application (included application documents, study proposals, and ethics approval of the institutional review board) of the current insurance data. The authors of the present study had no special access privileges in accessing the data which other interested researchers would not have.

## Supplementary Materials

The following supporting information can be downloaded at: https://www.mdpi.com/article/10.3390/jcm11061490/s1, Table S1: Characteristics of stroke patients received surgery with general and neuraxial anesthesia (before matching); Table S2: Risk of postoperative complications and mortality in stroke patients received surgery with general and neuraxial anesthesia (before matching); Table S3: The association between postoperative adverse events and general anesthesia stratified analysis by the characteristics of stroke.
