# Supplementary material for: Complications and Mortality after Surgeries in Patients with Prior Stroke Who Received General and Neuraxial Anesthesia: A Propensity-Score Matched Study
Source: J Clin Med. 2022 Mar 9;11(6):1490. doi: 10.3390/jcm11061490 (PMC8949151; doi:10.3390/jcm11061490)
Supplement: Supplementary file 1 [file jcm-11-01490-s001.zip › jcm-1592052-supplementary.pdf]

Table S1. Characteristics of stroke patients received surgery with general and neuraxial anesthesia (before matching).

	NA (n=21287)		GA (n=14862)		*p*-value
Sex	n	(%)	N	(%)	<0.0001
Female	7167	(33.7)	6341	(42.7)	
Male	14120	(66.3)	8521	(57.3)	
Age, years					<0.0001
20-29	133	(0.6)	124	(0.8)	
30-39	355	(1.7)	350	(2.4)	
40-49	699	(3.3)	923	(6.2)	
50-59	2204	(10.4)	1969	(13.3)	
60-69	4078	(19.2)	3142	(21.1)	
70-79	7307	(34.3)	4812	(32.4)	
≥80	6511	(30.6)	3542	(23.8)	
Low income					0.0403
No	20416	(95.9)	14188	(95.5)	
Yes	871	(4.1)	674	(4.5)	
Volume of hospital					<0.0001
Low	9578	(45.0)	2818	(19.0)	
Medium	6677	(31.4)	6462	(43.5)	
High	5032	(23.6)	5582	(37.6)	
Types of surgery					<0.0001
Skin	0	(0.00)	4	(0.03)	
Musculoskeletal	9305	(43.7)	6340	(42.7)	
Peripheral vascular	54	(0.3)	190	(1.3)	
Digestive	3740	(17.6)	2145	(14.4)	
Kidney, ureter, bladder	4886	(23.0)	4374	(29.4)	
Delivery, CS, abortion	249	(1.2)	49	(0.3)	
Others	3053	(14.3)	1760	(11.8)	
Number of hospitalizations					<0.0001
0	8290	(38.9)	5151	(34.7)	
1	6267	(29.4)	4206	(28.3)	
2	3096	(14.5)	2252	(15.2)	
≥3	3634	(17.1)	3253	(21.9)	
Number of emergency visits					<0.0001
0	6913	(32.5)	4415	(29.7)	

1	5493	(25.8)	3801	(25.6)	
2	3394	(15.9)	2582	(17.4)	
≥3	5487	(25.8)	4064	(27.3)	
Coexisting medical conditions					
Hypertension	9917	(46.6)	6448	(43.4)	<0.0001
Diabetes	5511	(25.9)	3994	(26.9)	0.0364
Hyperlipidemia	1116	(5.2)	747	(5.0)	0.3599
Mental disorders	5689	(26.7)	4150	(27.9)	0.0118
Ischemic heart disease	3746	(17.6)	2386	(16.1)	0.0001
Heart failure	1344	(6.3)	947	(6.4)	0.8230
COPD	3315	(15.6)	2031	(13.7)	<0.0001
Liver cirrhosis	509	(2.4)	511	(3.4)	<0.0001
Renal dialysis	434	(2.0)	483	(3.3)	<0.0001
Alcohol-related illness	458	(2.2)	482	(3.2)	<0.0001
Parkinson's disease	1389	(6.5)	818	(5.5)	<0.0001
Traumatic brain injury	1977	(9.3)	1479	(10.0)	0.0346

COPD, chronic obstructive pulmonary disease; CS: cesarean section; GA, general anesthesia; NA, neuraxial anesthesia.

Table S2. Risk of postoperative complications and mortality in stroke patients received surgery with general and neuraxial anesthesia (before matching).

	NA (n=21287)		GA (n=14862)		Outcome risk
Postoperative outcomes	Events	%	Event	%	OR (95% CI)†
30-day in-hospital mortality	100	0.5	133	0.9	2.22 (1.68-2.94)
Postoperative complications					
Pneumonia	801	3.8	843	5.7	1.82 (1.64-2.03)
Septicemia	927	4.4	1215	8.2	2.03 (1.84-2.23)
Acute renal failure	301	1.4	347	2.3	1.56 (1.32-1.84)
Pulmonary embolism	21	0.1	29	0.2	2.12 (1.17-3.84)
Urinary tract infection	2963	13.9	2028	13.7	1.01 (0.95-1.08)
Deep wound infection	84	0.4	73	0.5	1.39 (0.99-1.94)
Acute myocardial infarction	64	0.3	58	0.4	1.58 (1.09-2.31)
Postoperative bleeding	106	0.5	139	0.9	1.78 (1.36-2.34)
ICU stay	930	4.4	2269	15.3	4.37 (4.01-4.76)
Medical expenditure, USD‡	2139±1712		3423±3980		p<0.0001
Length of hospital stay, days‡	7.2±7.7		11.1±13.8		p<0.0001

CI, confidence interval; GA, general anesthesia; NA, neuraxial anesthesia; OR, odds ratio.

†Adjusted for all covariates listed in Table 1.

‡Mean±SD

Table S3. The association between postoperative adverse events and general anesthesia stratified analysis by the characteristics of stroke.

	Adverse events†‡	
	NA (n=4903)	GA (n=4903)
	OR (95% CI)‡	OR (95% CI)‡
Hemorrhage stroke	1.00 (reference)	2.08 (0.68-6.35)
Ischemic stroke	1.00 (reference)	1.84 (1.36-2.49)
Other stroke	1.00 (reference)	1.74 (1.54-1.98)
Stroke in preoperative 1-6 month	1.00 (reference)	1.69 (1.30-2.21)
Stroke in preoperative 7-12 month	1.00 (reference)	1.80 (1.35-2.41)
Stroke in preoperative 13-24 month	1.00 (reference)	1.86 (1.48-2.33)
Stroke in preoperative 25-60 month	1.00 (reference)	1.72 (1.41-2.12)
No use of anti-hypertensive drug	1.00 (reference)	1.70 (1.45-1.99)
Use of anti-hypertensive drug	1.00 (reference)	1.85 (1.55-2.22)
No use of statins use	1.00 (reference)	1.75 (1.51-2.03)
Use of statins	1.00 (reference)	1.81 (1.48-2.21)
No anticoagulant drug use	1.00 (reference)	1.70 (1.49-1.93)
Anticoagulant drug use	1.00 (reference)	2.01 (1.53-2.66)
No rehabilitation	1.00 (reference)	1.65 (1.44-1.91)
Rehabilitation	1.00 (reference)	2.03 (1.63-2.55)
No hemiplegia	1.00 (reference)	1.75 (1.54-1.99)
Hemiplegia	1.00 (reference)	1.75 (1.31-2.36)
No pressure ulcer	1.00 (reference)	1.79 (1.57-2.01)
Pressure ulcer	1.00 (reference)	1.27 (0.75-2.17)
No dementia	1.00 (reference)	1.81 (1.59-2.07)
Dementia	1.00 (reference)	1.52 (1.15-2.02)

CI, confidence interval; GA, general anesthesia; NA, neuraxial anesthesia; OR, odds ratio.

†Adverse events included with 30-day in-hospital mortality, pneumonia, septicemia, acute renal failure, pulmonary embolism, deep wound infection, postoperative bleeding.

‡Adjusted for all covariates listed in Table 1.
